# Co-utilization of glucose and xylose for the production of poly-β-hydroxybutyrate (PHB) by *Sphingomonas sanxanigenens* NX02

**DOI:** 10.1186/s12934-023-02159-2

**Published:** 2023-08-27

**Authors:** Yue Ming, Guoqiang Li, Zhuangzhuang Shi, Xin Zhao, Yufei Zhao, Ge Gao, Ting Ma, Mengmeng Wu

**Affiliations:** Key Laboratory of Molecular Microbiology and Technology, College of Life Sciences, Ministry of Education, Nankai University, 300071 Tianjin, PR China

**Keywords:** Poly-β-hydroxybutyrate, Co-utilization, Glucose, Xylose, Corn straw

## Abstract

**Background:**

Poly-β-hydroxybutyrate (PHB), produced by a variety of microbial organisms, is a good substitute for petrochemically derived plastics due to its excellent properties such as biocompatibility and biodegradability. The high cost of PHB production is a huge barrier for application and popularization of such bioplastics. Thus, the reduction of the cost is of great interest. Using low-cost substrates for PHB production is an efficient and feasible means to reduce manufacturing costs, and the construction of microbial cell factories is also a potential way to reduce the cost.

**Results:**

In this study, an engineered *Sphingomonas sanxanigenens* strain to produce PHB by blocking the biosynthetic pathway of exopolysaccharide was constructed, and the resulting strain was named NXdE. NXdE could produce 9.24 ± 0.11 g/L PHB with a content of 84.0% cell dry weight (CDW) using glucose as a sole carbon source, which was significantly increased by 76.3% compared with the original strain NX02. Subsequently, the PHB yield of NXdE under the co-substrate with different proportions of glucose and xylose was also investigated, and results showed that the addition of xylose would reduce the PHB production. Hence, the Dahms pathway, which directly converted D-xylose into pyruvate in four sequential enzymatic steps, was enhanced by overexpressing the genes *xylB*, *xylC*, and *kdpgA* encoding xylose dehydrogenase, gluconolactonase, and aldolase in different combinations. The final strain NX02 (Δ*ssB*, pBT*xylBxylCkdpgA*) (named NXdE II) could successfully co-utilize glucose and xylose from corn straw total hydrolysate (CSTH) to produce 21.49 ± 0.67 g/L PHB with a content of 91.2% CDW, representing a 4.10-fold increase compared to the original strain NX02.

**Conclusion:**

The engineered strain NXdE II could co-utilize glucose and xylose from corn straw hydrolysate, and had a significant increase not only in cell growth but also in PHB yield and content. This work provided a new host strain and strategy for utilization of lignocellulosic biomass such as corn straw to produce intracellular products like PHB.

## Background

Societal and environmental concerns are increasingly prominent due to the excess usage of non-biodegradable plastics with the increasing global population. Researchers have attempted several methods to solve this issue but few of them achieved desired results [1]. Therefore, there is an urgent need for safe and environmental-friendly novel materials to replace traditional plastics to solve a series of environmental issues [2]. In this situation, polyhydroxyalkanoates (PHAs), the only polyesters that are completely synthesized biologically, stand out due to their excellent features equivalent to petroleum-based plastics [3]. Polyhydroxybutyrate (PHB) is the most common type of PHAs and has been studied extensively [4]. PHB is synthesized intracellularly as a carbon source storage when the cell grows under environmental stress conditions such as low levels of nitrogen, oxygen, or ions, such as phosphates or sulfates [5, 6]. Reportedly, there are more than 300 microorganisms that can synthesize PHB, among which the most representative species including *Ralstonia eutropha*, *Alcaligenes latus*, *Aeromonas hydrophila*, *Bacillus* spp., and *Pseudomonas putida* [7, 8]. In the great mass of native PHB-accumulating species, a β-ketothiolase (encoded by *PhaA*) firstly condenses two acetyl-CoA to acetoacetyl-CoA, which then undergoes reduction by an NADPH-dependent reductase (encoded by *PhaB*) that produces 3-hydroxybutyryl-CoA [9, 10]. Finally, PHB is synthesized through the polymerization of 3-hydroxybutyryl-CoA by PHB synthase (encoded by *PhaC*) [11, 12].

The mechanical properties of PHB, such as bending modulus and tensile strength, are similar to polypropylene, and can be completely biodegradable [13]. Due to these excellent properties, PHB is used in packaging materials, bags, containers, and disposable items like cups and diapers [13–15]. It is also used either in surgical materials or as a biodegradable carrier for long-term dosages of drugs and insecticides/fertilizers [13, 15]. However, a major problem for the widespread production and commercialization of PHB is its high production cost as compared with conventional synthetic polymers [16, 17]. Raw material cost constitutes more than half the overall cost of production of biopolymer, of which about 70-80% is the carbon source used as a feedstock for the microorganism growth and biopolymer production [15]. It is necessary to find a very competitive carbon source to reduce its cost [18]. Lignocellulosic biomass (LCB), which is mainly composed of lignin (15-20%), cellulose (40-50%), and hemicellulose (25-30%), is the most abundant feedstock [19]. LCB can be converted into simple fermentable sugars, such as glucose, xylose, and arabinose, which can be utilized as substrates for the fermentative production of biodegradable plastic PHB [20]. Recent researches have been focused on utilizing agricultural waste to produce PHB [21]. *R*. *eutropha* is a well-known strain for PHB production from LCB. After fermentation on rice paddy straw as the single carbon source, the maximum PHB production was reported to be 11.42 g/L [22]. Dahman and Ugwu achieved 15.3 g/L biomass accumulation and 65% PHB content using wheat straw [23]. Rahman et al. tested the PHB accumulation by recombinant *Escherichia coli* in wastewater microalgae after hydrolysis and obtained 32% PHB content [24]. Kenaf biomass, another broadly available LCB, was utilized for PHB production and a relatively high PHB accumulation of 10.10 g/L was achieved after 36 h of fermentation [25]. Later, Soto et al. reported an integrated biorefinery concept and achieved a higher PHB accumulation of 12.10 g/L with a content of 80.1% per CDW using wheat straw [26]. In addition, switchgrass, date seed, and sugarcane bagasse inferred media were also used for PHB production [27, 28]. Therefore, it is a feasible and low-cost method for bioconversion of these lignocellulosic wastes to PHB [29, 30].

Although lignocellulosic biomass provides a novel strategy to decrease the high production cost of PHB production, the co-utilization of lignocellulosic hydrolyzed sugars, such as glucose and xylose, is another problem to be solved [31]. In nature, most microorganisms have carbon catabolite repression (CCR) and preferably utilize glucose, which is a huge bottleneck in maximizing the conversion of lignocellulosic biomass into value-added products [32]. The screening of biomass-utilizing strains that can utilize both glucose and xylose without the CCR is one of the best strategies [33]. *S*. *sanxanigenens* NX02 is a kind of gram-negative bacterium which could utilize glucose and xylose synergistically to produce various valued products such as exopolysaccharide and PHB since the lack of vital genes encoding specific enzymes of the phosphotransferase system (PTS), which could facilitate CCR [34]. Additionally, we found three xylose metabolic pathways in NX02: XI pathway, Dahms pathway, and Weimberg pathway. The characteristic of the XI pathway is the presence of xylose isomerase and xylulokinase. The intermediate metabolite xylulose will be phosphorylated to xylulose-5-phosphate and subsequently flown into the PP pathway [35]. The Dahms pathway and Weimberg pathway are oxidative pathways that are initiated with the oxidation of xylose to xylonate. The xylonate is then catalyzed into 2-keto-3-deoxy-xylonate, which is dehydrated to α-ketoglutaric semialdehyde in the case of the Weimberg pathway. The α-ketoglutaric semialdehyde will be oxidized to 2-oxoglutarate, which is converted in the tricarboxylic acid (TCA) cycle. In the case of the Dahms pathway, 2-keto-3-deoxy-xylonate is split by an aldolase into pyruvate and glycolaldehyde, and the former will be directly converted in the TCA cycle [36]. These three xylose metabolic pathways further ensure the synergistic utilization of glucose and xylose in the strain. In this work, *S*. *sanxanigenens* NX02 was studied to produce PHB efficiently. An engineered strain NXdE II was constructed through the optimization of the PHB biosynthetic pathway, and it could produce 21.49 ± 0.67 g/L PHB by co-utilizing glucose and xylose of corn straw total hydrolysate (CSTH) with a content of 91.2% CDW. This work showed that improvement of both cell growth and metabolic flux towards product precursor were vital for synthesis of intracellular products such as PHB. Moreover, the strain constructed here provided a new platform for the utilization of lignocellulosic biomass to produce other pyruvate-derived products.

## Results and discussion

### Blocking the production of exopolysaccharide for improving PHB production

**Fig. 1:**
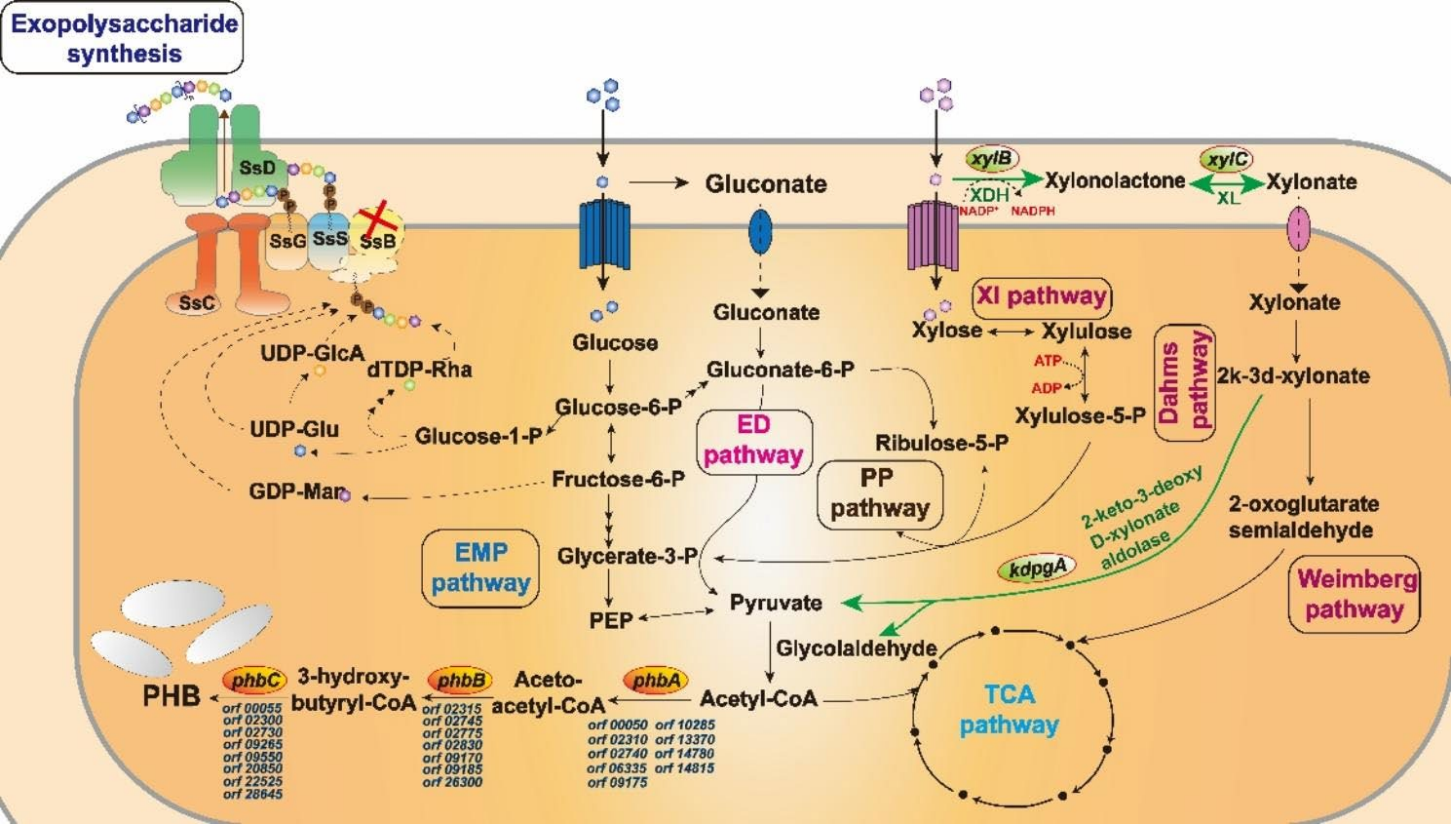
Overview of vital carbon metabolism pathways (glucose metabolism, xylose metabolism, exopolysaccharide synthesis, and PHB synthesis) in NX02. Arrows indicate the pathway steps encoded by the adjacent labeled gene products. Black arrows indicate the native pathways. Green arrows indicate the overexpressed pathways by addition of copies of native enzymes. Dotted black arrows indicate pathways abolished by deletion of the first glycosyltransferase (*ssB*). The white oval granules represent the product PHB. *Orf 00050*, *orf 02310*, *orf 02740*, *orf 06335*, *orf 09175*, *orf 10285*, *orf 13370*, *orf 14780*, and *orf 14815* represent genes encoding isoenzyme of β-ketothiolase; *Orf 02315*, *orf 02745*, *orf 02775*, *orf 02830*, *orf 09170*, *orf 09185*, and *orf 26300* represent genes encoding isoenzyme of acetoacetyl-CoA reductase; *Orf 00055*, *orf 02300*, *orf 02730*, *orf 09265*, *orf 09550*, *orf 20850*, *orf 22525*, and *orf 28645* represent genes encoding isoenzyme of PHB synthase. Abbreviations: EMP, Embden-Meyerhof-Parnas; ED, Entner-Doudoroff; PP, pentose phosphate; TCA, tricarboxylic acid; *phbA*, encoding β-ketothiolase; *phbB*, encoding NADPH-dependent acetoacetyl-CoA reductase; *phbC*, encoding PHB synthase; *xylB*, encoding xylose dehydrogenase; *xylC*, encoding xylonolactonase; *kdpgA*, encoding 2-keto-3-deoxy D-xylonate aldolase

The precursor of PHB synthesis is acetyl-CoA, which is catalyzed by pyruvate dehydrogenase complex. Pyruvate was formed mainly from the Embden-Meyerhof-Parnas (EMP) pathway, pentose phosphate (PP) pathway, and Entner-Doudoroff (ED) pathway [37]. However, in strain NX02, the branch pathway towards glucose-1-phosphate, which would be ultimately converted to precursors of exopolysaccharide synthesis existing at the glucose-6-phosphate node, caused the carbon loss towards PHB synthesis. Thus, the relationship between PHB synthesis and exopolysaccharide synthesis is competition in strain NX02 (Fig. 1).

**Fig. 2:**
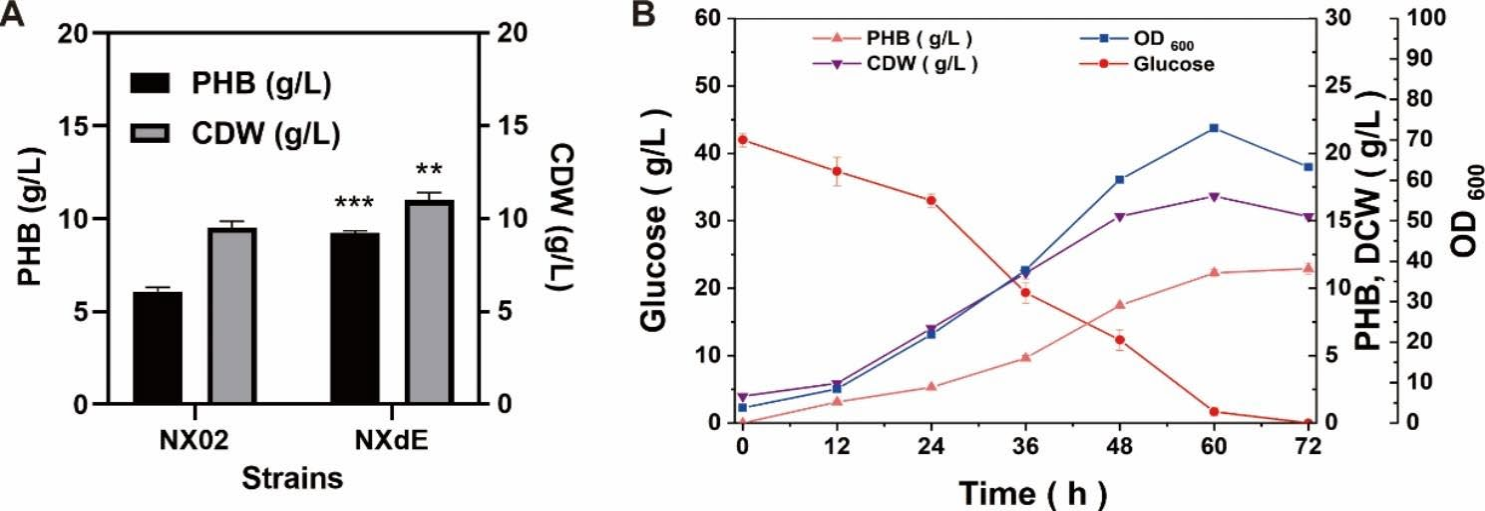
Comparison of fermentation using glucose as a sole carbon source by strain NX02 and NXdE. (**A**) Comparison of PHB yield and cell dry weight of shake flask fermentation in strain NX02 and NXdE; (**B**) Performance of batch fermentation in a 5 L fermenter by engineered strain NXdE. “**” indicates the significant differences (p-value<0.05) of cell dry weight between NX02 and NXdE, and “***” indicates the significant differences (p-value<0.01) of PHB yield between NX02 and NXdE

To improve PHB production, the metabolic pathway of exopolysaccharide synthesis was interrupted to accumulate more acetyl-CoA. Glucose-1-phosphate was involved in the formation of cell walls, and blocking the pathway from glucose-6-phosphate to glucose-1-phosphate might affect the growth and division of bacterium. Thus, gene *ssB* encoding the first glycosyltransferase responsible for polysaccharide biosynthesis was selected to be deleted by homologous recombination, resulting in strain NXdE (Fig. 1). To test the effect of deleting gene *ssB* on PHB production, the shake flask fermentations of strain NX02 and NXdE were performed and the cell dry weight and PHB production were detected. As shown in Fig. 2A, NX02 produced 6.08 ± 0.23 g/L PHB with a content of 63.7% CDW, and NXdE produced 9.24 ± 0.11 g/L PHB with a content of 84.0% CDW. Compared with NX02, the PHB production of NXdE was significantly increased by 76.3%. The results indicated that PHB production had a great improvement by blocking its competitive exopolysaccharide synthesis pathway.

To gain insight into the scale-up possibility of PHB production by strain NXdE, batch cultivation was conducted in a 5 L autoclavable fermenter and the results are shown in Fig. 2B. The strain NXdE obtained 15.32 ± 0.19 g/L cell dry weight and produced 11.45 ± 0.43 g/L PHB at 72 h, which were further increased by 23.9% compared with flask fermentation. According to our previous study, NX02 could produce 14.88 ± 0.83 g/L exopolysaccharide and 6.08 ± 0.23 g/L PHB in 5 L autoclavable fermenters under the same condition [37]. Both NX02 and NXdE had no glucose left at the end of the fermentation. Notably, the strain NXdE had an excellent sugar conversion rate compared with the original strain NX02 in terms of producing PHB. The high PHB production in NXdE may be due to its multi-copied key genes related to PHB biosynthesis. There are eight copies of *phbC*, seven copies of *phbB*, and nine copies of *phbA* in the genome of *S. sanxanigenens* NXdE (shown in Fig. 1) [38]. In recent years, some researchers isolated a novel *Sphingomonas* sp. from argan soil that can produce PHB. They studied the PHB production from argan seeds waste by this strain and the putative PHB produced was 1.92 g/L [39]. However, the PHB yield of *S*. *sanxanigenens* NXdE was 11.45 g/L, which was the highest reported yet among the *Sphingomonas* genus [37].

### Utilization of different proportions of glucose and xylose for PHB production

**Fig. 3:**
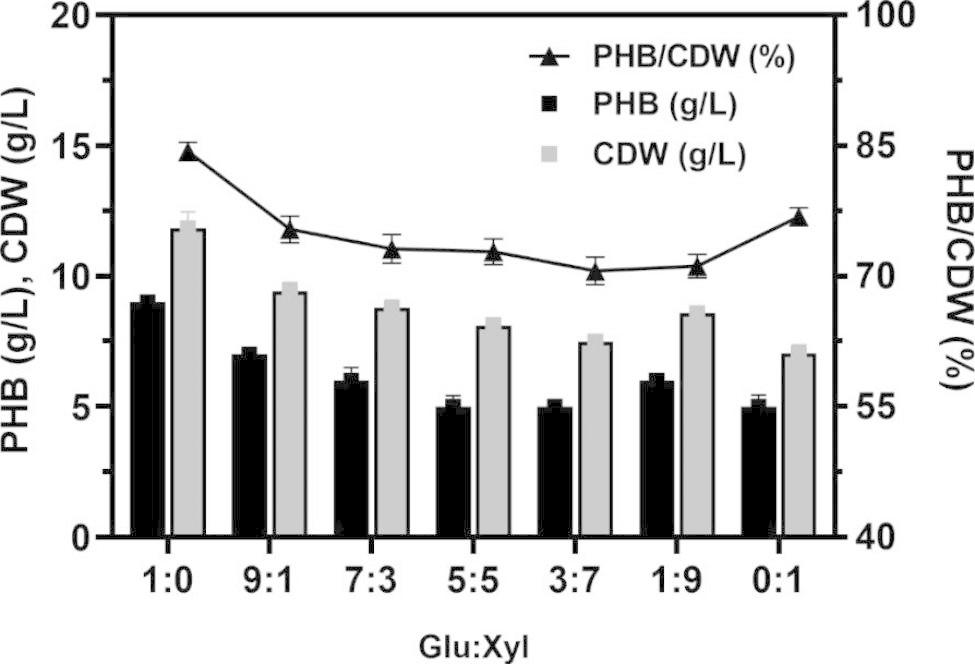
The shake flask fermentations of strain NXdE under different proportions of glucose and xylose

The strain NX02 can naturally utilize glucose and xylose simultaneously to produce bio-products [38], and its derivative strain NXdE also has this property. Based on this feature, we tested the possibility of strain NXdE co-utilizing glucose and xylose to produce PHB. The flask fermentation of NXdE under different proportions of glucose and xylose (the total amount of sugar mixture was 40 g/L) was performed. Results showed that fermentations using glucose and xylose as co-substrates were not as good as that of using glucose alone, and with the increase of xylose ratio, the cell dry weight and PHB production showed a basically decreasing trend. E.g., the PHB production was decreased to 5.00 ± 0.03 g/L from 9.20 ± 0.29 g/L and the cell dry weight was decreased to 7.48 ± 0.27 g/L from 11.83 ± 0.62 g/L, which were decreased by 45.7% and 36.8%, respectively (Fig. 3). It was obvious that PHB yield was reduced with the decrease of cell dry weight, and this phenomenon was common for intracellular products [40]. Moreover, the PHB content showed a slight downward trend with the increase of xylose ratio in fermentations using a mixed carbon source (Fig. 3). We speculated that the decrease of PHB production with the increase of xylose ratio was mainly due to the decrease of bacterium quantity caused by lower energy and precursor supply from xylose metabolism. Theoretically, 1 mol glucose can be converted to 2 mol pyruvate through the glycolysis pathway, and pyruvate can be used in the synthesis of PHB and enter the TCA pathway to provide a mass of energy. One mol xylose will form 1 mol pyruvate through the XI pathway and Dahms pathway while part of xylose will directly come into the TCA pathway through the Weimberg pathway. Thus, 1 mol xylose could only be converted into less than 1 mol pyruvate, which caused the decrease of energy and precursor supply from xylose metabolism (Fig. 1). Moreover, the deficiency of precursors was also a potential reason considering the PHB content that from 76.1 to 71.1% showed a decreasing trend with the increase of xylose ratio (Fig. 3).

### Enhancing Dahms pathway for improvement of PHB yield from xylose

**Fig. 4:**
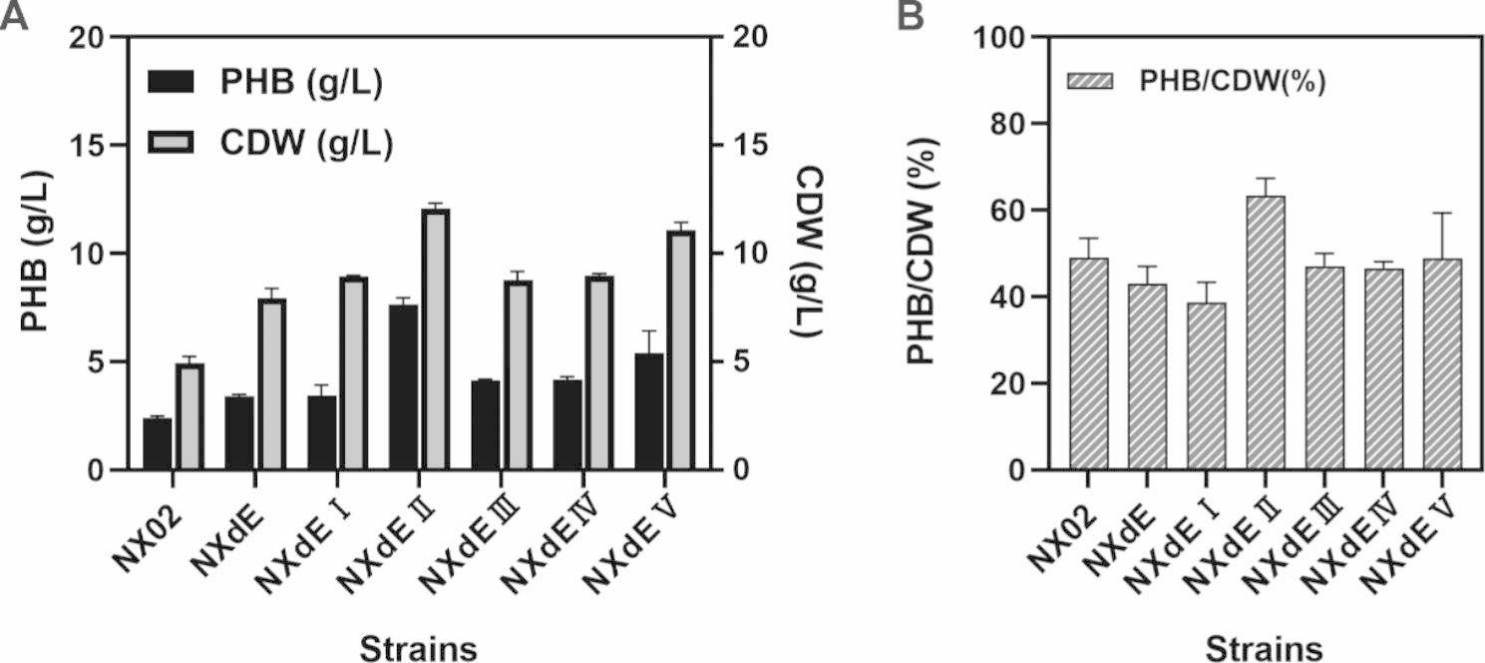
The shake flask fermentations using xylose as a sole carbon source by parent strain NX02 and six engineered strains. (**A**) The PHB yield and CDW of seven strains using xylose as a sole carbon source; (**B**) The PHB content of seven strains using xylose as a sole carbon source

Lignocellulose such as straw contains high levels of the suitable fermentable sugar D-xylose, the second-most abundant carbohydrate in nature after glucose [41]. To decrease the cost of production and implement the high conversion of lignocellulose into biodegradable and environmental-friendly polymer PHB, it is necessary to enhance the utilization of xylose by the engineered strain NXdE. Firstly, we tested the PHB performance with xylose as a sole carbon source between engineered strain NXdE and parent strain NX02. The results showed that the PHB production of NXdE was also increased from 2.41 ± 0.09 g/L to 3.41 ± 0.09 g/L and the cell dry weight was increased from 4.93 ± 0.31 g/L to 7.93 ± 0.45 g/L under the condition of xylose fermentation (Fig. 4A). Compared with NX02, the PHB production and CDW were increased by 41.5% and 60.9%, respectively.

As shown in Fig. 1, pyruvate is a key metabolite linking the TCA, glycolysis pathway, and xylose metabolism, and is also consumed through PHB synthesis. Therefore, it is necessary to choose a faster metabolic pathway from D-xylose to pyruvate for more efficient PHB synthesis. In this situation, the Dahms pathway was enhanced, which directly converted D-xylose into pyruvate in four sequential enzymatic steps: D-xylose is firstly oxidized by D-xylose dehydrogenase (encoded by gene *xylB*) to D-xylonolactone. Subsequently, D-xylonolactonase (encoded by gene *xylC*) catalyzed D-xylonolactone to D-xylonate, which then is converted to an intermediate, 2-keto-3-deoxyd-xylonic acid, by xylonate dehydratase (encoded by gene *xylD*). Lastly, 2-keto-3-deoxyd-xylonate is oxidized into pyruvate and glycolaldehyde by 2-keto-3-deoxy D-xylonate aldolase (encoded by gene *kdpgA*) [42]. It has been reported that introduction of the *Caulobacter crescent* genes *xylB* and *xylC* allowed the establishment of the Dahms pathway in *E*. *coli*. However, the engineered strain had poor growth because the carbon flux of xylose was flown mainly to the Dahms pathway, which led to reduced ATP availability and poor glycolysis [43, 44]. Based on the research above, the native genes *xylB* and *xylC* of strain NX02 were overexpressed (Fig. 1). The resulting strain was NXdE I, and the shake flask fermentation results showed that the CDW of NXdE I (8.93 ± 0.06 g/L) had a 12.6% increase but the PHB production (3.45 ± 0.48 g/L) had no significant change compared with the control strain NXdE (3.41 ± 0.09 g/L PHB and 7.93 ± 0.45 g/L CDW), which were quite different from the report. This might be caused by the existence of the Weimberg pathway which can enter the TCA cycle and supplement the energy for cell growth. Next, the native gene *kdpgA* encoding 2-keto-3-deoxy D-xylonate aldolase, the key enzyme that could catalyze 2-keto-3-deoxy-xylonate to pyruvate and glycolaldehyde of the Dahms pathway, was overexpressed to increase the amount of precursor pyruvate for improved PHB production (Fig. 1). Other three isoenzymes of aldolase from *E*. *coli* MG1655, which were encoded by *yagE*, *yjhH*, and *garL* severally were also overexpressed to explore the most appropriate isozyme [45, 46]. The resulting strain named NXdE II, NXdE III, NXdE IV, and NXdE V, respectively. Additionally, the enzyme xylonate dehydratase of the Dahms pathway as a shared pathway of the Weimberg pathway was not overexpressed in case excessive carbon flux will flow into the Weimberg pathway followed by the TCA cycle leading to a metabolic overflow. The shake flask fermentations of four engineered strains (NXdE II, NXdE III, NXdE IV, and NXdE V) were performed with xylose as a sole carbon source to explore the ability of PHB production. After overexpressing the three genes *xylB*-*xylC*-*kdpgA*, both PHB production and cell dry weight of strain NXdE II were increased to 7.65 ± 0.31 g/L and 12.07 ± 0.25 g/L drastically, which were increased by 124.3% and 52.2% respectively compared with NXdE I (Fig. 4A). On the other hand, the increased cell dry weight and PHB production were probably due to the increased pyruvate provided from Dahms pathway. In theory, 1 mol xylose can be converted into 1 mol pyruvate through both Dahms pathway and XI pathway, but the routes are different. In the case of Dahms pathway, the xylose is firstly dehydrogenated to xylonolactone and release a NADPH. Then, the xylonolactone is catalyzed into pyruvate by three steps without energy consumption [36]. In the case of XI pathway, the xylose is firstly catalyzed to xylulose by xylose isomerase. The resulting xylulose is subsequently phosphorylated to xylulose-5-phosphate and this step will consume ATP as energy. After that, the xylulose-5-phosphate will enter into the PP pathway. In summary, compared with XI pathway which will consume lots of energy and have a long series of reactions, the Dahms pathway takes less energy and is a faster route for synthesis of pyruvate. The more pyruvate also improved the energy productivity of TCA pathway (Fig. 1), which was better for cell growth. However, the results of three isoenzymes were not good enough compared with the native aldolase of NX02 (Fig. 4A). NXdE III, NXdE IV, and NXdE V produced 4.12 ± 0.06, 4.18 ± 0.13, and 5.39 ± 1.03 g/L PHB, respectively. In addition, the PHB/CDW value of NXdE II was about 63.33% and it was the highest among all seven strains (Fig. 4B).

**Fig. 5:**
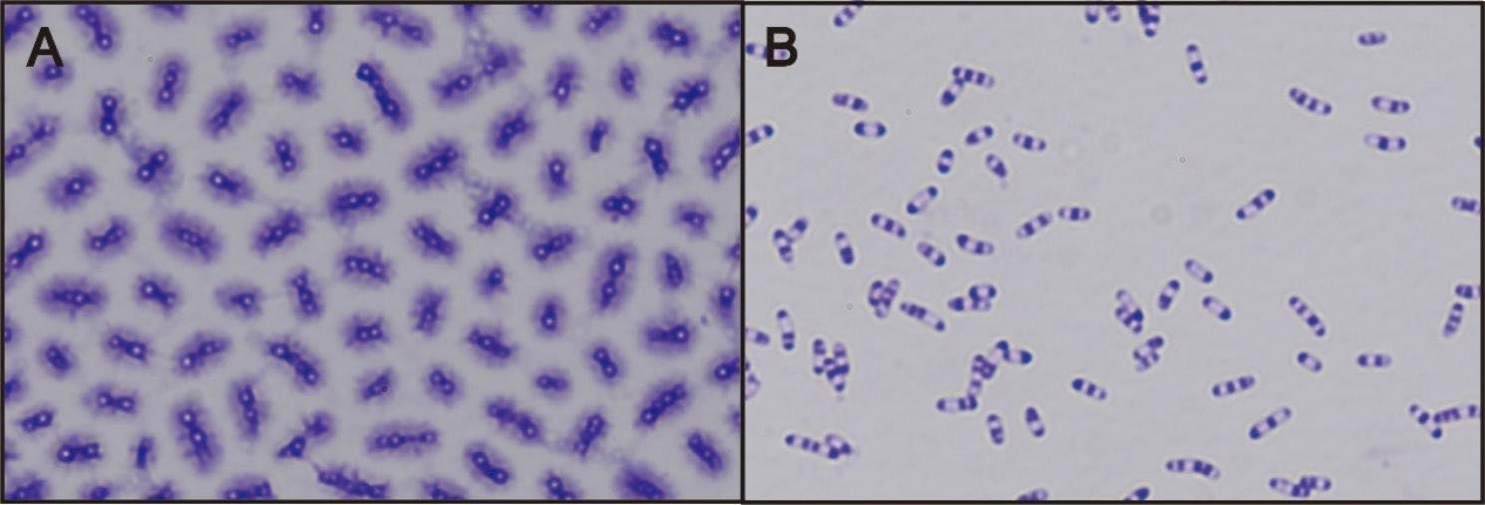
The microscopic observation of strain NX02 (**A**) and NXdE (**B**) at the end of fermentation using xylose as a sole carbon source

**Fig. 6:**
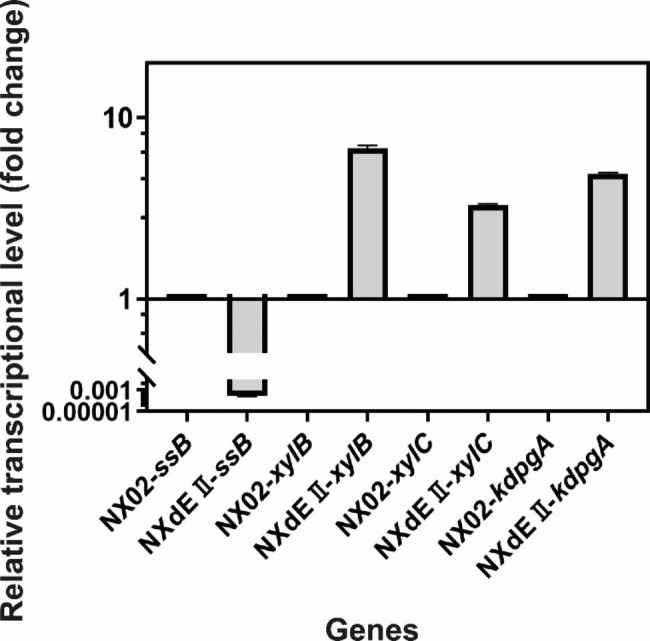
Relative transcriptional levels of genes *ssB*, *xylB*, *xylC*, and *kdpgA* in strain NX02 and NXdE II cultivated to exponential phase

The PHB content can also be observed simply using an optical microscope (Fig. 5). After crystal violet staining, the PHB granules were hard to be stained and it was white while other parts of the cells would be purple. It is obvious that strain NX02 could coproduce PHB and exopolysaccharide (fibrous) while strain NXdE II only produced PHB granules and the PHB granules are larger than those in NX02. The relative gene transcription levels of strain NXdE II were compared with parent strain NX02 through RT-PCR analysis (Fig. 6), which indicated that the gene *ssB* has been knocked down and all the genes related to enhancing the Dahms pathway have been successfully overexpressed. The relative transcription levels of gene *xylB*, *xylC*, and *kdpgA* were increased by 6.77-fold, 3.27-fold, and 4.86-fold respectively. Therefore, NXdE II was chosen for further research.

### Batch and fed-batch fermentations of strain NXdE II using glucose and xylose to produce PHB

**Fig. 7:**
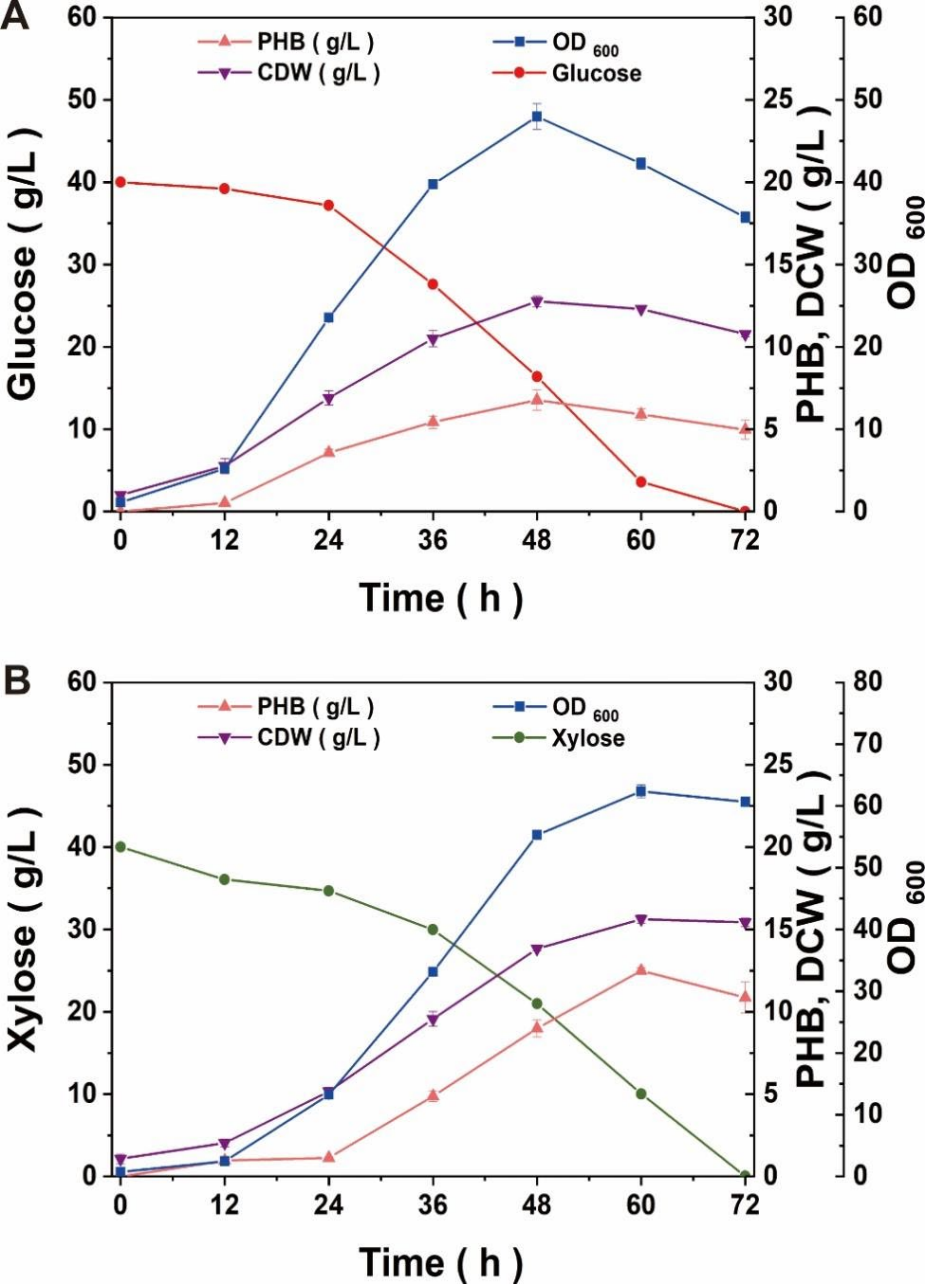
Batch fermentation performance of strain NXdE II using glucose (**A**) or xylose (**B**) as a sole carbon source in a 5 L fermenter

The batch fermentations with glucose or xylose as carbon sources were performed to investigate the growth and fermentation of the engineered strain NXdE II. The results of batch fermentations are shown in Fig. 7. When glucose was used as a carbon source, the strain reached the peak of growth at 48 h and produced 6.77 ± 0.61 g/L PHB with a content of 53.0% CDW (Fig. 7A). The highest yield of PHB in NXdE II was 0.29 ± 0.02 g/g glucose at 48 h. It is obvious that PHB production in glucose by engineered strain enhancing the Dahms pathway was decreased compared with the original strain (Figs. 2B and 7A). This phenomenon might be due to that the strategy of enhancing the Dahms pathway caused an imbalance of glucose and xylose metabolism and the cell growth and PHB synthesis was disturbed. The fermentation results using xylose as a carbon source showed that the strain reached the peak of growth at 60 h and produced 12.50 ± 0.18 g/L PHB with a content of 79.9% CDW (Fig. 7B). The highest yield of PHB in NXdE II was 0.31 ± 0.01 g/g xylose at 60 h. The PHB production of NXdE II through batch fermentation using xylose as a sole carbon source was increased by 63.4% compared with flask fermentation. This result verified that the engineered strain NXdE II could grow and ferment well in xylose.

**Fig. 8:**
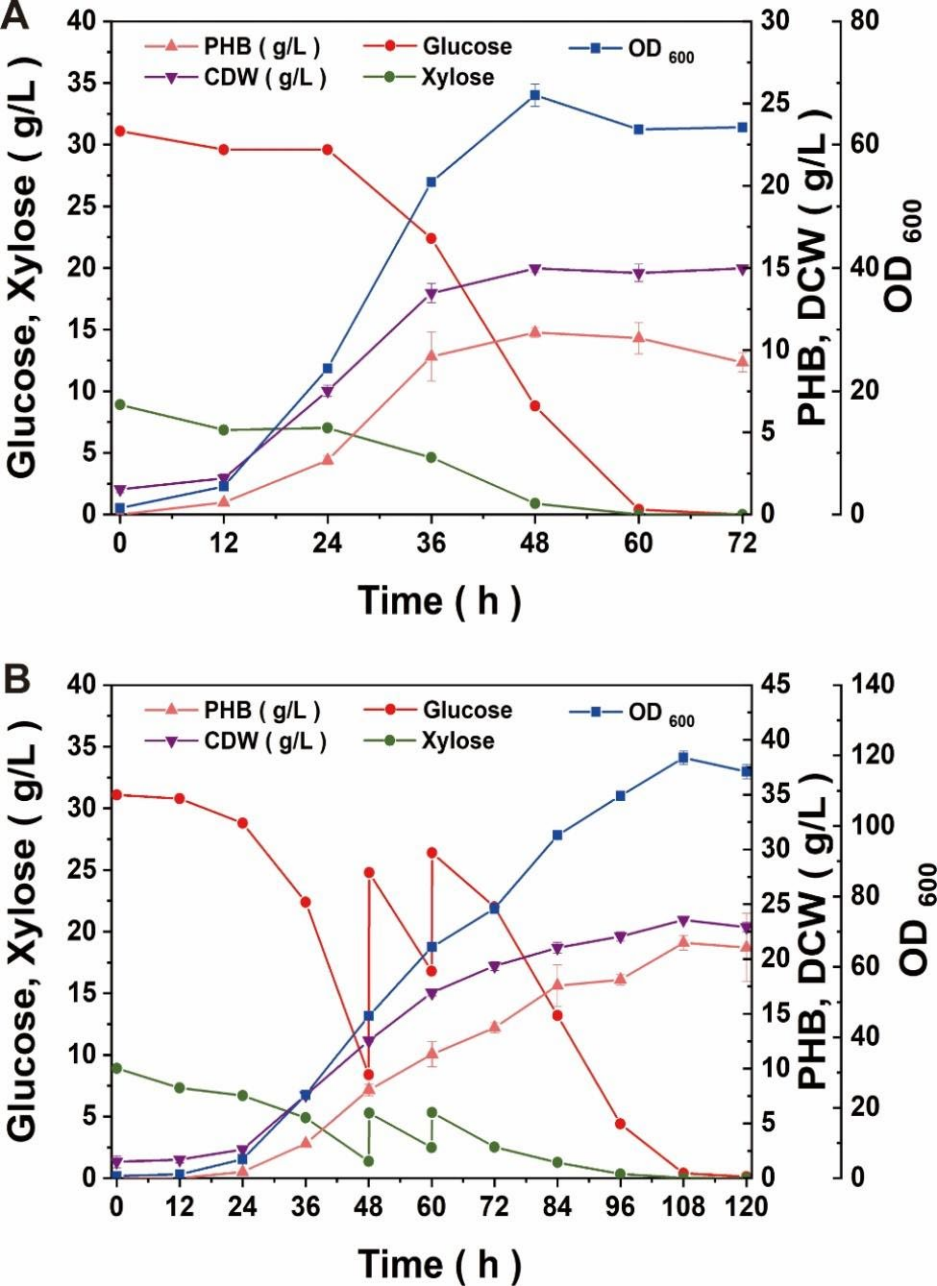
Batch fermentation performance of strain NXdE II using corn straw total hydrolysate as a carbon source in 5 L fermenters. (**A**) batch fermentation; (**B**) fed-batch fermentation feeding corn straw total hydrolysate

Based on the constructed engineered strain NXdE II, corn straw total hydrolysate (CSTH) was selected to characterize the PHB production potential [34]. The detailed method of preparation of corn straw total hydrolysate preparation of CSTH was indicated in our previous work [34] and herein a brief introduction was given in Materials and Methods part. The CSTH obtained containing 52.6 g/L glucose, 15.1 g/L xylose, and 1.8 g/L arabinose. The arabinose was not considered in this article due to its low content. The fermentation results using 40 g/L CSTH as a carbon source showed that the strain reached the peak of growth at 48 h and produced 11.07 ± 0.30 g/L PHB with a content of 73.9% CDW (Fig. 8A). The yield of PHB in batch fermentation using CSTH of NXdE II was 0.37 ± 0.01 g/g sugar, which was 1.28-fold higher than batch fermentation using glucose and 1.19-fold higher than batch fermentation using xylose. It can be seen from the results above that the engineered strain enhancing the Dahms pathway has achieved significant improvement in the mixed sugar fermentation of glucose and xylose. On one hand, the fermentation period of strain NXdE II was 24 h shorter than strain NXdE. The shorter production period means lower costs of manufacturing production. On the other hand, the PHB yield of strain NXdE II using CSTH (11.07 ± 0.30 g/L) reached the level of strain NXdE using glucose as a sole carbon source (11.45 ± 0.43 g/L) statistically. Moreover, it can be seen from the fermentation curve (Fig. 8A) that the proportion of glucose and xylose in the CSTH could be consumed properly by the strain at the same time. This phenomenon proved that the metabolic balance of strain NXdE II was more appropriate for the co-utilization of glucose and xylose in CSTH. In addition, strain NXdE II produced 21.49 ± 0.67 g/L PHB with a content of 91.2% CDW through fed-batch fermentation using CSTH as a carbon source and feeding CSTH (Fig. 8B). The yield of PHB in fed-batch fermentation using CSTH of NXdE II was 0.30 ± 0.01 g/g sugar. In summary, the resulting strain NXdE II produced 11.07 ± 0.30 g/L PHB with a yield of 0.37 ± 0.01 g/g sugar in the batch fermentation and 21.49 ± 0.67 g/L PHB with a yield of 0.30 ± 0.01 g/g sugar in the fed-batch fermentation. Therefore, corn straw total hydrolysate will be a better alternative substrate to produce PHB in NXdE II, which is a more promising PHB producing strain due to its capability of co-utilization of glucose and xylose efficiently.

## Conclusions

Through eliminating the competitive pathway of PHB production and enhancing the Dahms pathway, an engineered *S*. *sanxanigenens* NXdE II was obtained that could utilize xylose alone or mixed sugar (glucose and xylose) better. The final strain produced 21.49 ± 0.67 g/L PHB from corn straw total hydrolysate (CSTH) with a content of 91.2% CDW and the PHB yield was 0.30 ± 0.01 g/g sugar. This was a small advance in reducing manufacturing costs by utilizing lignocellulosic biomass to produce PHB. In the past decades, many research groups have studied PHB production with readily available alternative and inexpensive carbon sources such as lignocellulosic biomass [31]. Among them, some have achieved good results and the most commonly used strains include *C*. *necator* (named *R*. *eutropha* earlier), *Pseudomonas* spp., *Bacillus* spp., *E*. *coli*, and so on. Annamalai and Sivakumar study the PHB production using wheat bran hydrolysate as the carbon source with *C*. *necator* NCIMB 11,599 and produced cell dry weight, PHB and yield of 24.5 g/L, 62.5%, and 0.319 g/g sugar respectively [47]. Recently, Lee et al. constructed a coculture system of *C*. *necator* NCIMB 11,599 and *Bacillus* sp. SM01 can successfully utilize xylose of lignocellulosic biomass to produce PHB. This co-culture system can not only increase PHB production, but also overcome the limitation of sugar consumption [33]. However, the co-culture system does not solve the problem of co-utilizing glucose and xylose derived from lignocellulosic biomass fundamentally. About one year later, Lee et al. selected a novel *Loktanella* sp. SM43, which showed high utilization of both glucose and xylose, from a marine environment. *Loktanella* sp. SM43 could produce PHB using various lignocellulosic hydrolysates as feedstock and PHB production reached the highest at 3.66 ± 0.01 g/L when pine tree hydrolysates were used [31]. Therefore, the engineered strain NXdE II was excellent in co-utilizing glucose and xylose to produce considerable PHB without a co-culture system. The performance of NXdE II in producing PHB from CSTH also has an advantage over some others without genetic engineering in similar research.

In this work, we constructed an engineered strain to produce considerable PHB from lignocellulose waste. It is not only saving the raw material cost but also producing an excellent substitute that is “environment friendly” for petrochemically derived plastics. Furthermore, the engineered strain provided a new platform to produce other valuable chemicals from pyruvate.

## Materials and methods

### Strains, plasmids, media, and culture conditions

Bacterial strains and plasmids used in this study are listed in Table 1. *S*. *sanxanigenens* CGMCC 10,150 (strain NX02) and its engineered strains were cultured at 30 ℃ on NK medium containing (per liter): glucose 15.0 g, peptone 5.0 g, yeast extract, 1.0 g, beef powder 3.0 g, and agar 15.0 g (pH 7.0). 10% (v/v) NK culture was inoculated into the seed medium consisting of (per liter): sucrose 2.0 g, beef powder 5.0 g, peptone 3.0 g, and yeast extract 1.0 g (pH 7.5) [34] and the seed medium was cultivated at 200 rpm, 30 ℃. For PHB fermentation, strains were grown in a medium containing (per liter): carbon source (glucose, xylose, or the mixed sugars with different proportions of glucose and xylose) 40.0 g, peptone 1.0 g, K_2_HPO_4_ 1.5 g, FeSO_4_ 0.0001 g, MgSO_4_ 0.8 g, NaNO_3_ 1.5 g, sodium glutamate 4.0 g (pH 7.5). *E*. *coli* strains were cultured in Luria-Bertani medium. Antibiotics in this work were used at the following concentrations (µg/mL): kanamycin (Km; 25), tetracycline (Tc; 10), and chloramphenicol (Cm; 25). Beef powder, yeast extract, agar, peptone, and other chemicals were purchased from Solarbio Limited (Beijing, China). PHB standard was purchased from Sigma-Aldrich (America) [48].

**Table 1:** Strains and plasmids used in this study

Strain or plasmid	Genotype or phenotype	Source or reference
*Strains*		
*E. coli* S17	*RecA thi pro hsdR*^−^ M^+^ RP4, Sm^R^ Amp^R^ Kan^R^	This lab
NX02	Wild-type strain, Cm^R^	This work
NXdE	Strain NX02 (Δ*ssB*)	This work
NXdE I	Strain NX02 (Δ*ssB*) harboring vector pBT*xylBxylC*	This work
NXdE II	Strain NX02 (Δ*ssB*) harboring vector pBT*xylBxylC kdpgA*	This work
NXdE III	Strain NX02 (Δ*ssB*) harboring vector pBT*xylBxylCyagE*	This work
NXdE IV	Strain NX02 (Δ*ssB*) harboring vector pBT*xylBxylCyjhH*	This work
NXdE V	Strain NX02 (Δ*ssB*) harboring vector pBT*xylBxylCgarL*	This work
*Plasmids*		
pLO3	4937-bp suicide vector, tet^R^	
pLO3Δ*ssB*	pLO3 derivative carrying upstream and downstream fragment of *ssB*	This work
pBT	A vector from pBBR1mcs-2, and the promoter has been changed from P_*lac*_ to P_*tac*_	This work
pBT-*BC*	pBT derivative expressing *xylBC*, *xylBC* refers to *xylB*, and *xylC*	This work
pBT-*BCA*	pBT derivative expressing *xylBCA*, *xylBCA* refers to *xylB*, *xylC, and kdpgA*	This work

### Preparation of corn straw total hydrolysate (CSTH)

Firstly, the corn straw was dried and milled and followed by acid hydrolysis using 2.0% H_2_SO_4_ with a liquid/solid ratio of 10:1 (w/w) at 100 ℃ for 2 h. After that, the corn straw was cooled and adjusted the pH to 4.8 with Ca (OH)_2_. Then the cellulase and β-glucosidase were used to enzymatically hydrolyze the pretreated biomass at 45 ℃ and 150 rpm for 72 h. Finally, the CSTH was obtained by discoloring and multiple purifying [34].

### Construction of the engineered strains

The *ssB* gene in NX02 was inactivated by double-crossover homologous recombination. The upstream and downstream flanking sequences (approximately 1.0 kb) of *ssB* were spliced with linearized plasmid pLO3 using Gibson Assembly kit (E2611S, New England BioLabs) to form the recombinant plasmid pLO3Δ*ssB*, then it was transformed to *E. coli* S17 competent cell. The plasmid pLO3Δ*ssB* was transferred to *S. sanxanigenens* wild-type strain using biparental filter mating at 30 °C for 12 h on NK medium without antibiotics. The single crossover mutant was screened on NK medium with 25 µg/mL Cm and 10 µg/mL Tc at 30 °C. The knockout mutant NX02 (Δ*ssB*) was then isolated on NK medium of which glucose was instead of sucrose containing 25 µg/mL Cm, followed by PCR screening using the verification primers. For the sake of narrative, the strain NX02 (Δ*ssB*) was renamed NXdE in the following.

Plasmid pBT was derived from the broad host range constitutive expression vector pBBR1mcs-2 by replacing the weak *Plac* promoter with the strong *Ptac* promoter using PCR [48]. For gene overexpression, *xylB*, *xylC*, and *kdpgA* were cloned into vector pBT, separately or in pairs, to form plasmids pBT-*BC*, pBT-*BCA*. All overexpression vectors were individually transferred into the strain NXdE using conjugal transfer to construct the engineered strains shown in Table 1. For all engineered strains, RNA was extracted for reverse transcription and real-time quantitative PCR to detect transcription levels of knockout or overexpressed genes (Fig. 6). All primers used in the construction of engineered strains are shown in Table 2.

**Table 2:** All primers used in this study

Primer name	Primer sequence (5→3)
Primers for genetic manipulation	
pBTBC_F	GAAAGGGACGAGGATGGCGAGGAGCTCCAATTCGCCCTAT
pBTBC_R	GATCCCCAGCGACCAGGCGGCCCGGTACCTTCTCCTTA
pBTBCA_F	GTTTCGGGATTGAGGACCAGGGGAGCTCCAATTCGCCCTAT
BC_F	TAAGGAGAAGGTACCGGGCCGCCTGGTCGCTGGGGATC
BC_R	ATAGGGCGAATTGGAGCTCCTCGCCATCCTCGTCCCTTTC
BCA_R	CTTTCCCGGTGTCTCGTTGTGTCGCCATCCTCGTCCCTTTC
kdpgA_F	GAAAGGGACGAGGATGGCGACACAACGAGACACCGGGAAAG
kdpgA_R	ATAGGGCGAATTGGAGCTCCCCTGGTCCTCAATCCCGAAAC
Primers for RT-PCR	
RT16S_F	TCAAACGCTGGTAAGGTTCTGC
RT16S_R	GCGGCTCACTGGACTGGTATT
RTssB_F	CGTTCAGGATCTGCGGCAG
RTssB_R	ATCTGGCCCTGACGCTTGC
RTxylB_F	TGAATGCGTCGATCGAGGTT
RTxylB_R	GTAGAGGACGAGCTCGGGCA
RTxylC_F	TGTGGTTCGGCAGCATGGAC
RTxylC_R	GATATGCTTCACCGGCGTGC
RTkdpgA_F	CTTCACCCACCTCTCCGCC
RTkdpgA_R	GATCATCGCGGTCTGGGTG

### RNA extraction and (RT)-PCR analysis

The *S. sanxanigenens* strains shown in Table 1 were cultured in NK medium, and then collected when cultivated at 24 h. The crude total DNA-free RNA of *S. sanxanigenens* strains was extracted using the RNAiso Plus (Takara, Dalian, China) and RNAprep Pure Cell/Bacteria Kit (Tiangen, China). The cDNA was amplified using FastKing RT Kit (Tiangen, Beijing, China) with the total mRNA as the template. Samples were then analyzed using Agilent 6820 (Agilent, America) with RealMasterMix (SYBR Green I) (Tiangen, Beijing, China). Quantity real-time PCR amplification primers were listed in Table 2. For data analysis, the *16 S* gene was selected as the internal standard for normalization between samples, and three biological replicates were performed. The obtained data were analyzed by using the 2^−ΔΔCt^ method [11].

### Bioreactor fermentations

All engineered strains were grown in NK medium at 30 ℃ for about 24 h. 10% (v/v) NK culture was inoculated into the seed medium which was ready for bioreactor fermentation after being grown for 24 h. The batch cultivation was performed in 5 L quadruple autoclavable fermenters (Shanghai Bailun Biotechnology Co., Ltd., Shanghai, China), with a final working volume of 3.0 L. The temperature was set at 30 ℃ and the pH was maintained at 7.0 by adding NaOH or HCl liquid automatically [34]. During the initial 12 h, the agitation speed was set at 300 rpm, then adjusted to 500 rpm in order to increase the dissolved oxygen concentration as what is needed for strains growing and biosynthesizing is largely more than provided. The aeration rate was set at 1.0 *vvm* (air volume/culture volume/min).

### Microscopy and staining procedures

To observe the intracellular PHB granules, 10 µL of *S. sanxanigenens* fermentation broth was dropped and spread on a microscopy slide. After the liquid was dried naturally, an appropriate amount of crystal violet dye was added and retained for two minutes. Rinsing the crystal violet dye off gently with water and blotting up the water. For the detection, a light microscope with a 100× oil objective was used [49].

### Analysis of cell growth and sugar consumption

Cell growth was measured either as optical density (OD) at 600 nm with spectrophotometer BD1015 (BioDrop) or by measuring cell dry weight (CDW). To obtain CDW, 1.5 mL Eppendorf tubes were weighted priorly. Then 1.0 ml of fermentation broth samples were added into the weighed tubes and centrifuged at 12,000 rpm for 2 min. Tubes with samples were dried for 24 h at 60 °C and weighed [50].

Glucose measurement was done with the SBA-40 C biosensor (Key laboratory for biosensors of Shandong province). Samples were diluted 40 times in water and filtered with 0.22 μm syringe filter units. 25 µL of the filtrate was injected into the SBA-40 C biosensor to determine the glucose concentration. Xylose measurement was done using colorimetric micromethod with phloroglucinol. Place 1.0 mL of xylose solutions and 5.0 mL of phloroglucinol color reagent in digestion tubes and mix well. Heat all tubes for exactly 8 min at 100 ℃, then cool to room temperature in ice. After mixing, read the absorbances at 554 nm [51].

### PHB quantification

To determine the intracellular PHB content, 1.0 ml of cells were harvested by centrifugation at 12,000 rpm for 2 min [52]. The supernatants were removed, and cells were washed three times with distilled water. Each sample was subjected to methanolysis by heating at 100 ℃ for 300 min in a solution containing 1.0 ml chloroform, 30 µl sulfuric acid, 2 µl internal standard (benzoic acid), and 968 µl methanol. In addition, PHB purchased was treated similarly as a reference sample [50]. After the samples were cooled to room temperature, 1.0 ml aliquot of distilled water was added to the methyl ester solution and the mixtures were vortexed for 5 min. All the solutions were centrifugated at 5000 rpm for 5 min. The chloroform layer was carefully extracted and moved into 1.5 mL Eppendorf tubes. The samples were filtered using 0.22 μm syringe filter units and injected into gas chromatograph system (GC, Agilent 6890 N, USA) equipped with an HP-PONA column and a flame ionization detector (FID). The inlet temperature of the gas chromatograph was 200 °C, and nitrogen was supplied as a carrier gas at 2 mL/min. The initial oven temperature was 90 ℃ for 1 min, then rose to 150 ℃ at 8 ℃ /min for 10 min. The FID temperature was maintained at 250 ℃ during the operation [33].

## Data Availability

The datasets supporting the conclusions of this article are included within the article.
